# Preserved Linear Growth in Overweight or Obese Children Treated with Soagambi-Tang: A Real-World Clinical Study with Computational Analysis

**DOI:** 10.3390/ph19081257

**Published:** 2026-08-10

**Authors:** Yeon-Joo Yoo, Chae-Yeon Kang, Min-Seong Lee, Young-Min Ahn, Byung-Cheol Lee

**Affiliations:** 1 Department of Clinical Korean Medicine, Graduate School, Kyung Hee University, 26, Kyungheedae-ro, Dongdaemun-gu, Seoul 02447, Republic of Korea; alice98113@khu.ac.kr (Y.-J.Y.); cykang@khu.ac.kr (C.-Y.K.); min6691@khu.ac.kr (M.-S.L.); omdan@hanmail.net (Y.-M.A.); 2 Department of Nephrology & Endocrinology, Kyung Hee University Korean Medicine Hospital, Kyung Hee Medical Center, 23 Kyungheedae-ro, Dongdaemun-gu, Seoul 02447, Republic of Korea

**Keywords:** pediatric obesity, height velocity, herbal medicine, puberty-associated endocrine signaling, in silico analysis, real-world clinical study

## Abstract

**Background/Objectives**: Pediatric obesity is linked to adverse metabolic outcomes and altered growth trajectories. Conventional weight-reduction strategies often raise concerns regarding potential impairment of linear growth, highlighting the need for approaches that reduce adiposity while preserving physiological development. **Methods:** This real-world clinical study investigated children aged 7–16 years who were administered the herbal medicine Soagambi-tang for 2 weeks, followed by a follow-up evaluation 6 months post-treatment. Anthropometric measures, body composition, and growth velocity were evaluated. Network pharmacology and molecular docking analyses were employed to investigate potential molecular mechanisms. **Results**: Twenty-five participants were included. After treatment, the proportion of children classified as obese decreased from 60.0% to 36.0%, and 32.0% showed improvement in weight-status category without any worsening. Body fat percentage decreased consistently among participants with available body composition data, from 30.24 ± 4.52% to 25.95 ± 5.44% (*p* < 0.0001), and fat mass significantly decreased from 15.22 ± 5.60 kg to 14.28 ± 5.77 kg (*p* = 0.0496), whereas lean body mass increased from 36.12 ± 12.08 kg to 38.89 ± 12.70 kg (*p* < 0.001). BMI percentile significantly decreased from 93.7 ± 3.9 to 91.2 ± 6.1 (*p* = 0.008), while height z-score and height percentile significantly increased from 0.57 ± 0.99 to 0.77 ± 1.07 and from 64.5 ± 25.5 to 68.6 ± 25.0, respectively (both *p* < 0.001). Mean annual height velocity was 7.45 ± 2.43 cm/year, and height change was positively correlated with lean body mass change (r = 0.543, *p* = 0.005), reflecting a meaningful upward shift in growth trajectory. In silico network pharmacology and molecular docking analyses identified key bioactive compounds in Soagambi-tang that were predicted to be associated with pathways related to pediatric obesity and puberty-associated endocrine regulation. **Conclusions**: These findings suggest that Soagambi-tang treatment was associated with reduced adiposity and preserved linear growth in children with overweight or obesity. Given the study design and limited sample size, controlled prospective studies are required to confirm efficacy and safety.

## 1. Introduction

Pediatric obesity is associated with adverse cardiometabolic outcomes and disruptions [1] in normal growth trajectories [2]. In addition to increasing the risk of insulin resistance, dyslipidemia, and type 2 diabetes [3], excess adiposity during childhood has been implicated in alterations of linear growth patterns and pubertal timing through interactions with endocrine pathways, including the growth hormone–IGF-1 axis [4].

Despite the clinical importance, the management of pediatric obesity remains challenging [5]. Conventional strategies—such as caloric restriction, lifestyle modification, and pharmacotherapy—primarily target adiposity reduction but may inadvertently affect linear growth, particularly during critical developmental windows [6]. Insufficient caloric intake and reduced weight gain are well-established contributors to impaired linear growth, reflecting the energy-dependent nature of pediatric growth processes [7]. This raises a clinical concern that interventions aimed at improving metabolic health could interfere with normal growth processes. Whether adiposity reduction can be achieved without compromising linear growth in pediatric populations, however, remains insufficiently defined, reflecting a limitation of current therapeutic paradigms.

Emerging evidence suggests that body composition, rather than body weight per se, is a more relevant determinant of metabolic health. Accordingly, therapeutic approaches that reduce fat mass while preserving lean mass and supporting physiological growth may be more appropriate in children. Nonetheless, clinical evidence supporting the coexistence of adiposity reduction and maintained growth remains limited.

Multi-component phytochemical interventions have been proposed to modulate metabolic homeostasis through coordinated effects on lipid metabolism, energy balance, and inflammatory signaling pathways. While calorie-restriction-based approaches for pediatric obesity can be associated with temporary deceleration of height velocity during active weight reduction, traditional phytochemical medicine may offer a complementary strategy by modulating body composition, including reductions in fat mass and potential preservation of lean tissue, thereby supporting linear growth without imposing an energy deficit that could hinder growth. However, their potential to concurrently influence adiposity and growth in pediatric populations has not been systematically evaluated. Soagambi-tang is a multi-component herbal prescription used in Korean medicine for weight management and metabolic regulation in children. Soagambi-tang contains multiple phytochemically characterized herbal components, with representative constituents including ephedrine-type alkaloids from *Ephedra sinica* [8], cinnamaldehyde from *Cinnamomum cassia* [9], gingerols from *Zingiber officinale* [10], glycyrrhizin from *Glycyrrhiza uralensis* [11], paeoniflorin from *Paeonia lactiflora* [12], and amygdalin from *Prunus armeniaca*. Several of these constituents have been reported to influence energy expenditure, glucose and lipid metabolism, and inflammatory and metabolic signaling, providing a plausible multi-target basis for the metabolic regulatory properties attributed to the formulation. Given its combined herbal composition, it may exert multi-target effects on lipid metabolism, energy homeostasis, and endocrine-related pathways.

In this real-world clinical study, we examined children with overweight or obesity who underwent a Soagambi-tang for weight management. Changes in anthropometric parameters, body composition, and growth velocity were assessed, and potential mechanisms were explored using network pharmacology and molecular docking analyses. The findings indicate a pattern of reduced adiposity concurrent with preserved linear growth, suggesting a potentially distinct clinical phenotype in pediatric obesity and precocious puberty that warrants further investigation.

## 2. Results

### 2.1. Real-World Clinical Study

#### 2.1.1. Baseline Characteristics

**Figure 1:**
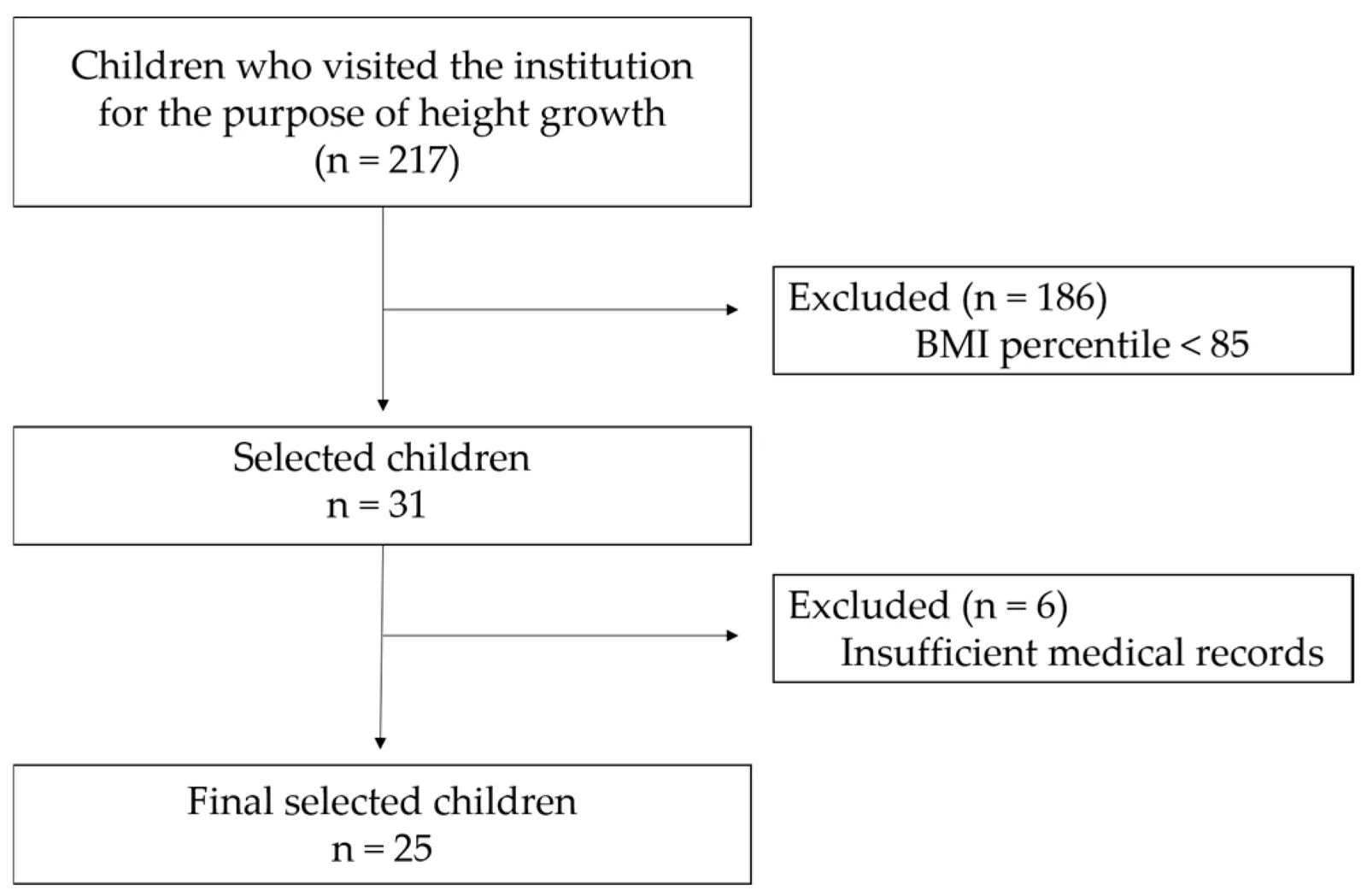
Flow diagram of study participants.

During the study period, 217 outpatients presented with concerns of insufficient height growth. Following application of the predefined eligibility criteria, 25 patients were included in the final analysis (Figure 1).

The study cohort comprised 17 boys and 8 girls, with a mean age of 10.52 ± 2.18 years. Mean follow-up duration was 5.19 ± 1.42 months. One female participant had reached menarche at baseline. Mean height, body weight, and body mass index (BMI) were 145.64 ± 13.22 cm, 49.00 ± 14.07 kg, and 23.11 ± 3.42 kg/m^2^, respectively. Body fat percentage was available in 23 participants, with a mean baseline value of 30.24 ± 4.52% (Table 1).

No participants received additional pharmacological or procedural obesity treatments during the study period. All participants received brief lifestyle guidance focused on reducing sugar-containing foods and beverages, which should be considered a potential co-intervention.

#### 2.1.2. Changes in Obesity-Related Outcome Measures

**Figure 2:**
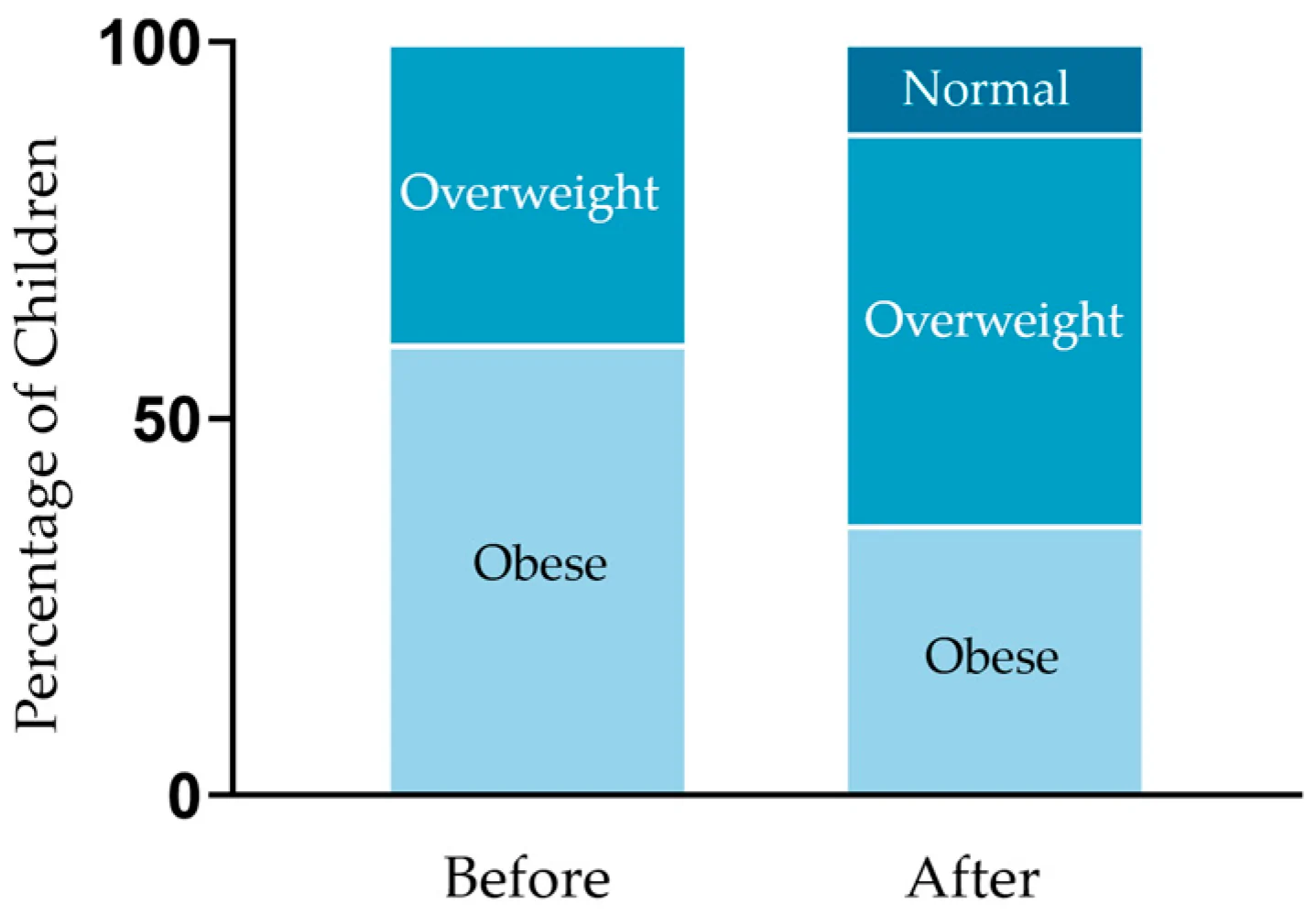
Changes in weight status category in the real-world clinical study.

Among the 25 children evaluated, the baseline distribution showed that 15 children were obese (60.0%) and 10 were overweight (40.0%), while none were classified as normal before treatment. After treatment, the proportion of obese children decreased to 9 children (36.0%), whereas 13 children (52.0%) were classified as overweight and 3 children (12.0%) reached the normal range. Overall, 8 of 25 children (32.0%) showed improvement in weight-status category: 1 obese child improved to normal, 5 obese children improved to overweight, and 2 overweight children improved to normal. The remaining 17 children (68.0%) stayed in the same category, and no child worsened to a higher weight-status category. These findings suggest that the treatment was associated with a shift toward lower obesity severity, particularly through a reduction in the proportion of obese children (Figure 2).

**Figure 3:**
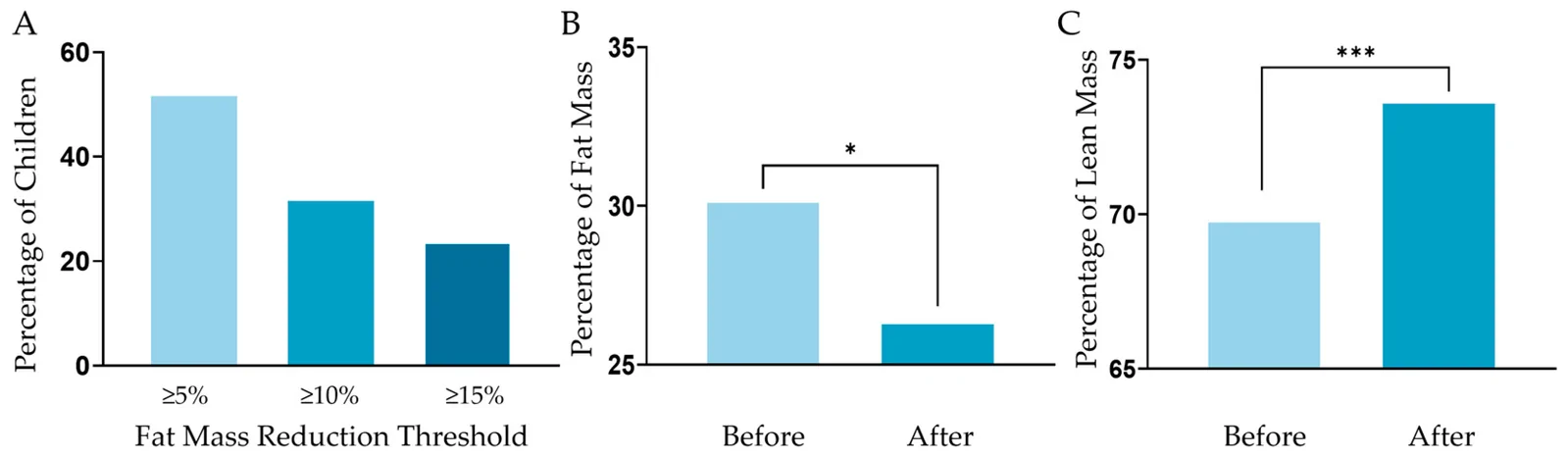
Changes in body composition. (**A**) Changes in fat mass reduction. (**B**) Changes in fat mass percentage (%). (**C**) Changes in lean mass percentage (%). Data are presented as mean ± SEM; * *p* < 0.05, *** *p* < 0.001.

Among the 23 participants with available body composition data, body fat percentage decreased consistently in all individuals. Mean body fat percentage declined from 30.24 ± 4.52% to 25.95 ± 5.44% (mean change −4.29 ± 3.19 percentage points; *p* < 0.0001). Fat mass decreased from 15.22 ± 5.60 kg to 14.28 ± 5.77 kg, showing a significant reduction (mean difference −0.94 ± 2.28 kg, *p* = 0.0496). Fat mass change percentage was −6.16 ± 14.83%, showing a statistically significant reduction (*p* = 0.0487). The proportions of children achieving ≥5%, ≥10%, and ≥15% reductions in fat mass were 52.0%, 32.0%, and 24.0%, respectively. In contrast, lean body mass significantly increased from 36.12 ± 12.08 kg to 38.89 ± 12.70 kg (mean difference + 2.77 ± 2.79 kg, *p* < 0.001). Lean body mass change percentage was +7.23 ± 6.41%, showing a statistically significant increase (*p* < 0.001). These findings suggest that body composition changes during the observation period were characterized not by simple weight reduction, but rather by a favorable shift toward increased lean mass with relative reduction in adiposity (Figure 3).

**Figure 4:**
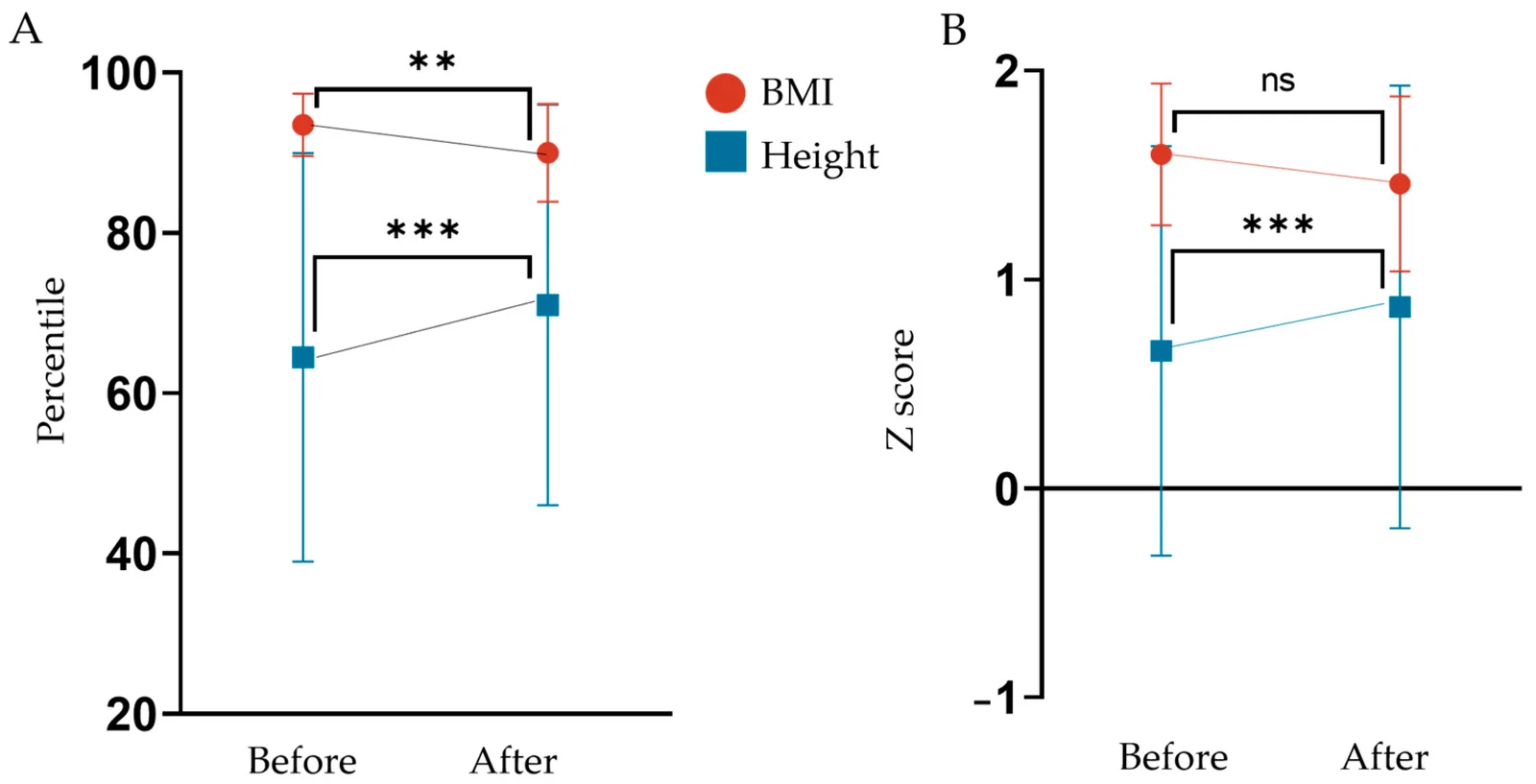
Changes in z-score and percentile. (**A**) Changes in BMI and height percentile. (**B**) Changes in BMI and height z-score. Data are presented as mean ± SEM; ** *p* < 0.01, *** *p* < 0.001.

BMI percentile decreased from 93.7 ± 3.9 to 91.2 ± 6.1 (mean difference −2.44 ± 4.22, Cohen’s d = −0.58, *p* = 0.008), and BMI z-score also decreased from 1.52 ± 0.44 to 1.41 ± 0.48 (mean difference −0.11 ± 0.29, Cohen’s d = −0.38, *p* = 0.059) (Figure 4).

#### 2.1.3. Changes in Height-Related Outcome Measures

**Figure 5:**
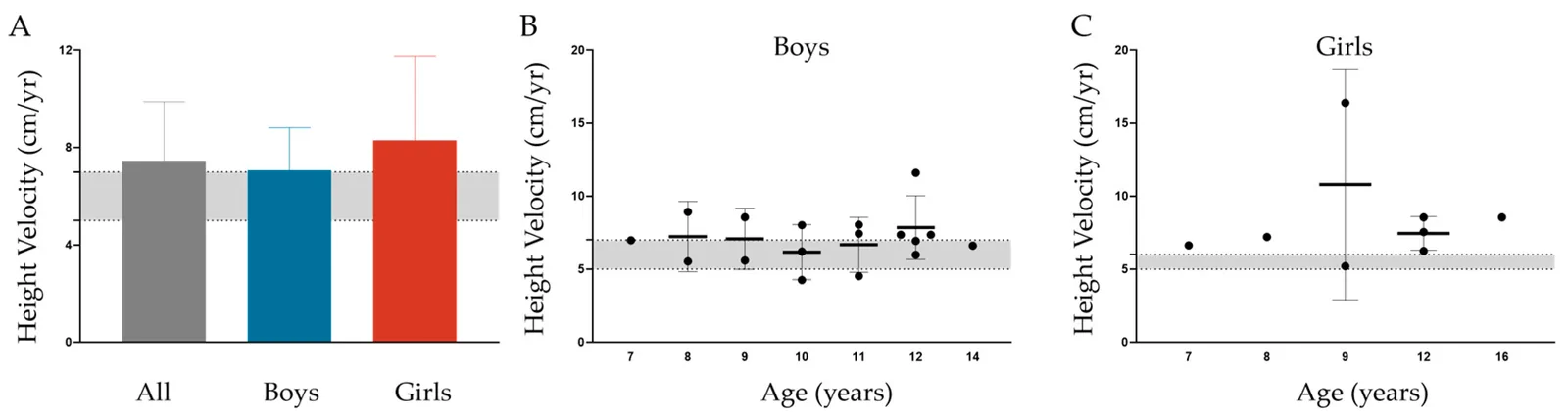
Height velocity of study participants. (**A**) Overall height growth rates stratified by sex. (**B**) Height growth rates of male participants according to age. Gray indicates the mean height growth rate, and data points represent the growth rates of individual participants. (**C**) Height growth rates of female participants according to age. Gray indicates the mean height growth rate, and data points represent the growth rates of individual participants.

Mean height increased from 145.64 ± 13.22 cm to 149.19 ± 13.31 cm, corresponding to a mean gain of 3.55 ± 1.0 cm over the observation period (*p* < 0.001). The mean annual height growth velocity of the cohort was 7.45 ± 2.43 cm/year, which was generally higher than the expected average growth velocity for school-aged children (approximately 5–6 cm/year). Overall height velocity in both male and female participants was comparable to or greater than the expected age- and sex-specific average values, 7.06 ± 1.76 cm/yr and 8.29 ± 3.46/yr, respectively (Figure 5). Growth velocities in children aged 11–12 years were particularly elevated, consistent with pubertal growth acceleration in some participants. While most values were within or above the expected range for age, a few cases showed exceptionally high growth velocities, especially a 9-year-old girl (16.4 cm/year) and a 16-year-old girl (8.55 cm/year). 

Height z-score significantly increased from 0.57 ± 0.99 to 0.77 ± 1.07 (mean difference + 0.20 ± 0.23, Cohen’s d = 0.88, *p* < 0.001), indicating a large effect on linear growth. Similarly, height percentile significantly increased from 64.5 ± 25.5 to 68.6 ± 25.0 (mean difference + 4.07 ± 4.91, Cohen’s d = 0.83, *p* < 0.001), suggesting an upward shift in growth trajectory (Figure 4). Collectively, these findings suggest that height progression improved while BMI-related indices remained stable or mildly decreased, reflecting a favorable change in growth balance and body composition rather than excessive weight gain relative to linear growth.

An exploratory age-stratified analysis was also performed. Concerning children less than 10 years of age, height z-score increased from 0.73 ± 1.02 to 0.85 ± 1.06 after treatment; however, this change was not statistically significant in the paired *t*-test (mean change, +0.12 ± 0.22; *p* = 0.133). In the non-parametric Wilcoxon signed-rank test, the increase reached statistical significance (*p* = 0.043), suggesting a tendency toward improved height z-score despite the small sample size. As for children 10 years and older, height z-score significantly increased from 0.62 ± 0.98 before treatment to 0.87 ± 1.09 after treatment, with a mean change of +0.25 ± 0.23. Paired *t*-test showed a statistically significant increase in height z-score (*p* = 0.00066), which was also confirmed by Wilcoxon signed-rank test (*p* = 0.00076).

#### 2.1.4. Correlation Between Outcome Measures

**Figure 6:**
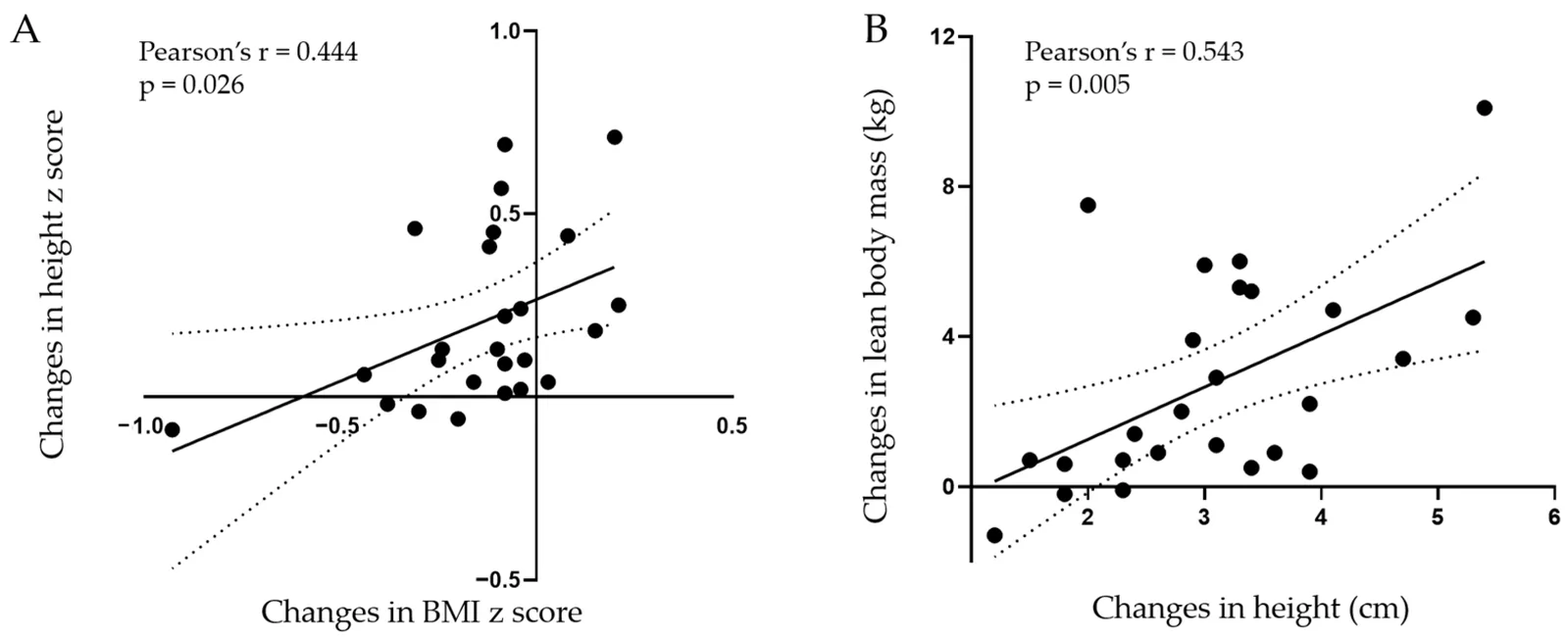
Correlation analyses of parameters. (**A**) Correlation between changes in BMI z-score and height z-score. (**B**) Correlation between changes in height and lean body mass. Filled circles represent individual participants. The solid line indicates the fitted linear regression line, and the dotted lines represent the 95% confidence interval of the regression estimate. Pearson’s correlation coefficient (r) and the corresponding *p* value are shown in each panel.

Pearson correlation analysis demonstrated a significant positive association between changes in BMI z-score and changes in height z-score (Pearson’s r = 0.444, *p* = 0.026), indicating that participants with greater increases in height z-score tended to show greater increases, or smaller decreases, in BMI z-score. Changes in height were also significantly and positively correlated with changes in lean body mass (Pearson’s r = 0.543, *p* = 0.005), suggesting that linear growth was accompanied by gains in lean mass (Figure 6). This correlation should be interpreted in the context of somatic growth, as increases in height and lean body mass may accompany stable or slightly increased BMI z-scores during normal maturation.

#### 2.1.5. Adverse Events

A retrospective real-world observational cohort study was conducted using longitudinal electronic medical records data extracted from the outpatient clinical database. Systematic audit of follow-up EHR entries revealed no documented clinically notable adverse events during Soagambi-tang treatment. In particular, no sympathomimetic reactions or clinically apparent cardiovascular symptoms, such as palpitations, were recorded. Furthermore, no patients required dose modification or treatment discontinuation due to adverse clinical events. Medication adherence was monitored by counting the remaining decoction packets at follow-up visits.

### 2.2. Results of Computational Analysis

#### 2.2.1. Results of Network Pharmacology Analysis

**Figure 7:**
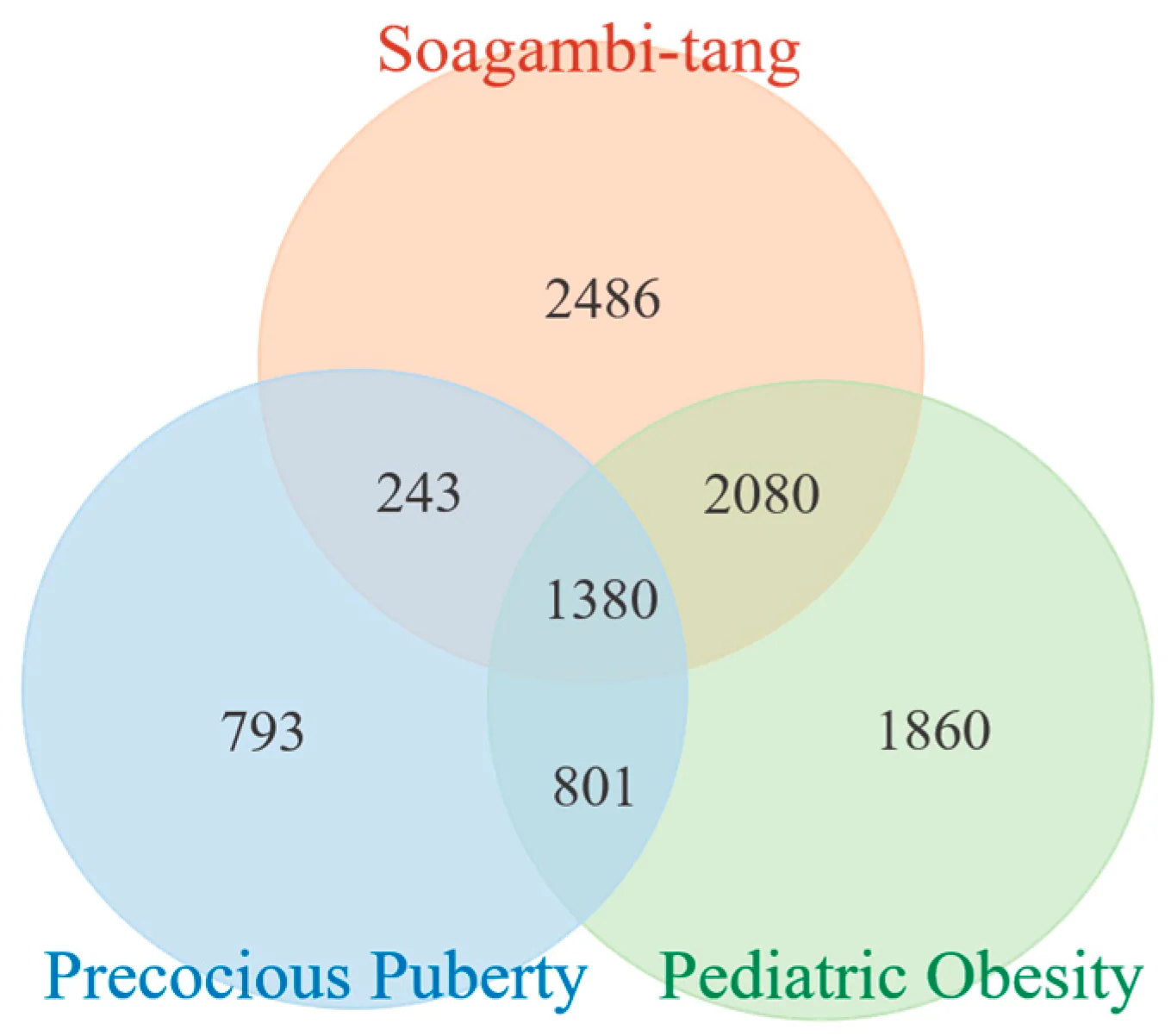
Venn diagram of overlapping GeneCards-derived targets among Soagambi-tang, precocious puberty, and pediatric obesity.

**Figure 8:**
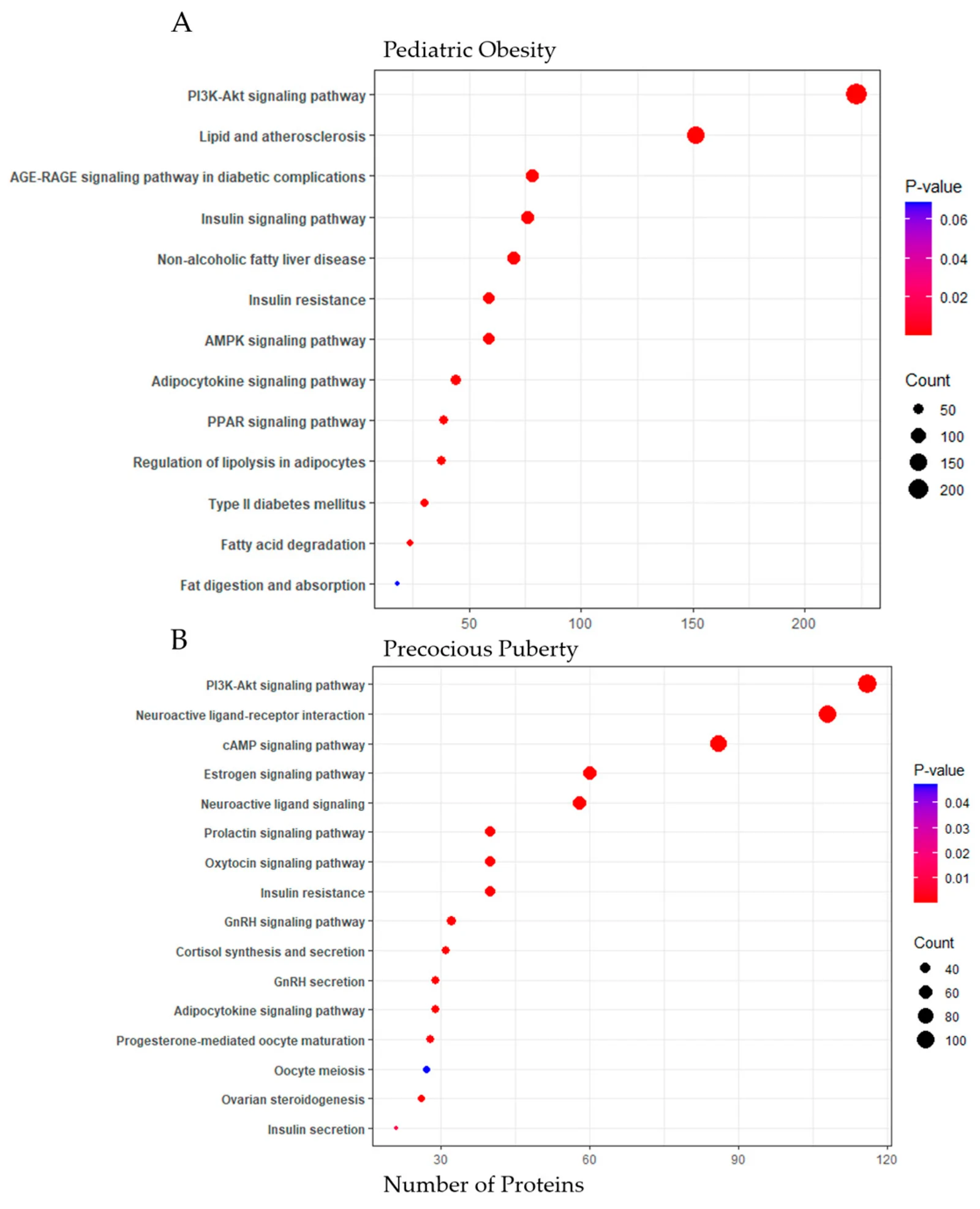
KEGG pathway enrichment of overlapping targets. (**A**) KEGG pathway between Soagambi-tang and pediatric obesity. (**B**) KEGG pathway between Soagambi-tang and precocious puberty. Dot size corresponds to the number of enriched genes, and dot color represents the −log10 (*p*-value).

According to the Venny analysis using GeneCards-derived targets, substantial overlap was observed among Soagambi-tang, precocious puberty, and pediatric obesity-associated genes. A total of 2181 genes were shared between precocious puberty and pediatric obesity, while 1623 genes overlapped between Soagambi-tang and precocious puberty, and 3460 genes overlapped between Soagambi-tang and pediatric obesity. Notably, 1380 genes were commonly shared among Soagambi-tang, precocious puberty, and pediatric obesity, suggesting that these overlapping genes may represent a putative target network linking Soagambi-tang, pediatric obesity, and precocious puberty-related pathways (Figure 7). From a network pharmacology perspective, obesity is increasingly recognized as a multifactorial disorder driven by interconnected signaling pathways rather than a single molecular target. KEGG pathway enrichment analysis indicated that Soagambi-tang is associated with key metabolic pathways, including insulin resistance, FOXO signaling, AGE–RAGE signaling in diabetic complications, and the insulin signaling pathway. In terms of precocious puberty, Soagambi-tang shared pathways related to growth hormone synthesis and endocrine development (Figure 8).

#### 2.2.2. Results of Molecular Docking Analysis

**Figure 9:**
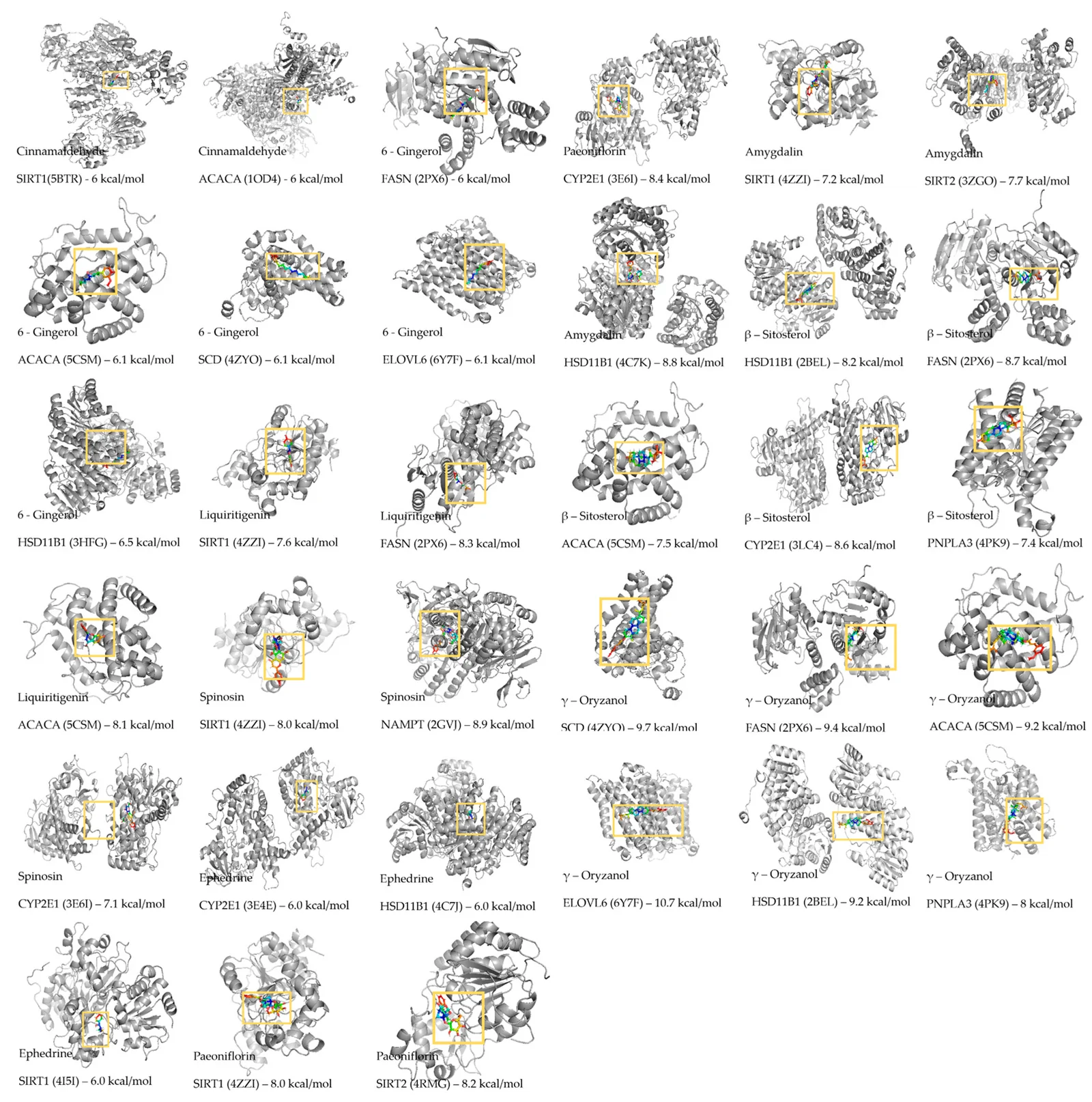
Representative docking conformations and predicted binding affinities of Soagambi-tang and pediatric obesity. Protein structures are shown as gray ribbons, while ligands are represented as colored sticks within the predicted binding pockets, highlighted by yellow boxes. The corresponding target protein, PDB ID, and binding affinity are indicated below each docking model. Binding energies are expressed in kcal/mol.

Concerning pediatric obesity, molecular docking analysis demonstrated that the investigated phytochemicals preferentially converge on two principal regulatory axes rather than acting on isolated targets. Notably, strong binding affinities were observed for enzymes involved in de novo lipogenesis, including ACACA, FASN, SCD, and ELOVL6, as well as for NAD^+^-dependent metabolic regulators such as SIRT1, SIRT2, and NAMPT (Figure 9). Compound class–specific interaction patterns were also identified. Sterol-derived compounds showed preferential binding to enzymes within the lipogenic pathway, suggesting coordinated modulation of fatty acid synthesis and lipid accumulation. In contrast, flavonoid and glycoside compounds exhibited higher affinities for NAD^+^-dependent regulators, indicating potential effects on cellular energy homeostasis and mitochondrial function. This complementary targeting profile supports a network-level mechanism in which distinct phytochemical classes exert convergent regulatory effects on metabolic pathways despite differing molecular interactions. These findings suggest coordinated modulation of lipogenesis and NAD^+^-dependent signaling rather than isolated compound-specific actions. Collectively, the results indicate that the observed metabolic effects may be mediated through integrated regulation of the ACACA–FASN–SCD–ELOVL6 lipogenic axis and the SIRT–NAMPT metabolic axis, representing a multi-pathway framework of metabolic control. These mechanistic insights provide biological plausibility for the observed dissociation between adiposity reduction and preserved linear growth.

**Figure 10:**
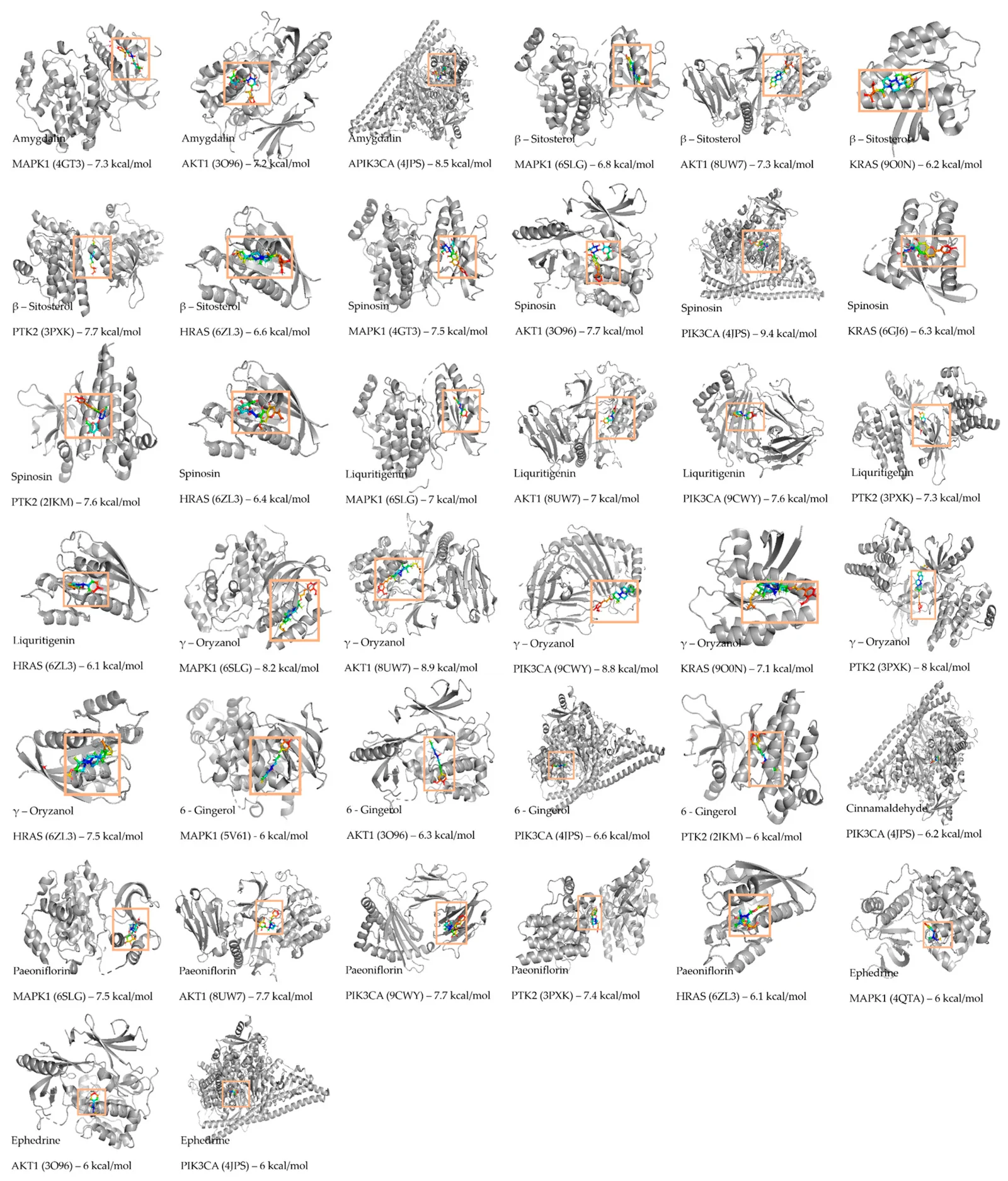
Representative docking conformations and predicted binding affinities of Soagambi-tang and precocious puberty. Protein structures are shown as gray ribbons, while ligands are represented as colored sticks within the predicted binding pockets, highlighted by yellow boxes. The corresponding target protein, PDB ID, and binding affinity are indicated below each docking model. Binding energies are expressed in kcal/mol.

Secondly, molecular docking analysis suggested that several phytochemical compounds may interact with target proteins implicated in precocious puberty–related signaling pathways as well. Among the tested compounds, γ-oryzanol showed consistently strong binding affinities with multiple targets, including MAPK1, AKT1, PIK3CA, and PTK2, with binding energies ranging from −8.0 to −8.9 kcal/mol. Spinosin and amygdalin also exhibited strong interactions with PIK3CA, showing binding affinities of −9.4 and −8.5 kcal/mol, respectively, while paeoniflorin demonstrated favorable binding to PIK3CA (−8.3 kcal/mol). These findings indicate that the phytochemical intervention may modulate key intracellular signaling pathways, particularly MAPK, PI3K–AKT, and PTK2-related pathways, which are involved in endocrine regulation, cellular growth, and metabolic signaling (Figure 10).

## 3. Discussion

In this real-world clinical study, Soagambi-tang treatment was associated with a favorable pattern of body recomposition in children with overweight or obesity. Although changes in BMI-related indices were modest and heterogeneous, body fat percentage decreased consistently and lean mass increased, while height-related indices, including height z-score and height percentile, improved during the observation period. Importantly, all participants continued to show linear growth, and estimated annualized height velocities were generally within or above age-appropriate physiological ranges. These findings suggest a treatment-associated pattern of body recomposition characterized by reduced adiposity and preserved or enhanced growth-related parameters, rather than nonspecific weight reduction. Given the observational design and absence of a control group, however, these changes should be interpreted as descriptive clinical observations.

Pediatric obesity is a multifactorial condition closely linked to cardiometabolic risk, ref. [13] altered body composition, and disturbances in endocrine regulation [14]. Recent clinical guidelines emphasize comprehensive lifestyle modification, including dietary, physical activity, and behavioral interventions, as the foundation of pediatric obesity management [15]. Pharmacotherapy may be considered as an adjunctive treatment in selected children and adolescents when sufficient improvement is not achieved with intensive lifestyle interventions [16]. The therapeutic landscape has expanded with the increasing use of glucagon-like peptide-1 receptor agonist. A recent systematic review and meta-analysis of randomized controlled trials reported significant improvements in body weight, BMI, glycemic parameters, and several cardiometabolic outcomes with GLP-1 receptor agonists in children and adolescents, although gastrointestinal adverse events were more frequent and available follow-up periods were relatively short [17]. These developments highlight the increasing availability of pharmacological options for pediatric obesity while underscoring the continued importance of evaluating long-term safety, growth, and developmental outcomes in this population. In children, excessive adiposity may also interact with pubertal timing and growth trajectories through pathways involving insulin signaling, leptin dynamics, inflammatory mediators, and the growth hormone–insulin-like growth factor-1 axis [18]. Therefore, weight management strategies in this population should aim not only to reduce adiposity but also to preserve normal growth and developmental potential.

The present findings may be clinically relevant in this context. In pediatric populations, weight-management strategies that markedly reduce energy intake may raise concerns regarding inadequate energy availability during critical periods of growth [19,20]. Therefore, interventions for pediatric obesity should ideally reduce excess adiposity while preserving lean mass and normal linear growth. The observed decrease in body fat percentage, together with increased lean body mass and improved height-related indices, suggests a potential shift toward relatively leaner growth. The positive correlation between changes in height and lean body mass suggests that these changes occurred concurrently; however, this exploratory association should be interpreted cautiously. A plausible explanation is that the multi-component phytochemical formulation exerts coordinated effects on lipid metabolism and energy homeostasis, enabling adiposity reduction while maintaining sufficient resources to support growth [21]. In addition, the overlap between pediatric obesity and precocious puberty-related target networks suggests that metabolic and endocrine pathways may be biologically interconnected. Because obesity-related insulin resistance, adipose tissue dysfunction, and chronic low-grade inflammation have been implicated in early pubertal activation, improvement in metabolic homeostasis may indirectly influence puberty-related signaling. However, pubertal status was not systematically assessed in this study; therefore, any interpretation regarding precocious puberty should be considered exploratory and hypothesis-generating.

Age-stratified analyses also showed numerical changes in height-related outcomes in both age groups. However, these subgroup analyses were exploratory, were based on small numbers of participants, and were performed without adjustment for multiple comparisons. Therefore, differences in the magnitude or statistical significance of changes between age groups should not be interpreted as evidence of age-specific treatment effects. Pubertal stage, baseline growth potential, treatment duration, and other unmeasured factors may also have contributed to the observed differences.

Collectively, the present data suggest that reductions in adiposity were observed with continued linear growth, while insufficient total energy availability may be the critical factor underlying growth attenuation. In this context, interventions that selectively target adiposity while preserving energy sufficiency may represent a more physiologically balanced approach to pediatric growth and metabolic health.

The in silico analyses provide preliminary mechanistic support for the observed clinical pattern. Network pharmacology suggested that Soagambi-tang-related targets were enriched in pathways associated with lipid metabolism, insulin signaling, endocrine regulation, and inflammatory responses. Molecular docking analyses further indicated that several phytochemical constituents showed predicted binding affinities toward proteins involved in de novo lipogenesis, including ACACA, FASN, SCD, ELOVL6, and PNPLA3, as well as NAD^+^-dependent metabolic regulators such as SIRT1, SIRT2, and NAMPT. These targets are broadly involved in lipid synthesis, mitochondrial function, substrate utilization, and cellular energy homeostasis. Among them, ACACA, FASN, SCD, and ELOVL6 form a functionally interconnected axis regulating de novo fatty acid synthesis and lipid handling, providing a more focused framework for interpreting the predicted interactions in relation to the observed weight-related effects. At the same time, because Soagambi-tang is a multi-component herbal formulation, the broader pattern of predicted interactions may reflect its potential to modulate multiple, interconnected metabolic targets rather than a single dominant molecular pathway.

The predicted interactions with PI3K–AKT and MAPK pathway-related proteins may also be relevant to the combined regulation of growth, metabolism, and pubertal signaling. In particular, compounds such as spinosin, amygdalin, paeoniflorin, and γ-oryzanol showed favorable predicted binding profiles toward targets including PIK3CA, AKT1, MAPK1, and PTK2. These pathways participate in intracellular growth signaling, insulin-related metabolic regulation, hypothalamic signaling, and reproductive endocrine processes. These predicted interactions support the possibility that Soagambi-tang may influence multiple metabolic and endocrine signaling networks, although direct biological activity remains to be experimentally confirmed.

In addition, individual herbal components of the formulation Soagambi-tang have been independently associated with metabolic regulatory effects. *Ephedra sinica* has been linked to appetite regulation and gut microbiota modulation [22], while *Cinnamomum cassia* [23] and *Zingiber officinale* [24] have demonstrated effects on glucose and lipid metabolism as well as inflammatory signaling. Other components, including *Glycyrrhiza uralensis* [25], and *Paeonia lactiflora* [26] have been implicated in AMPK activation and PI3K/AKT signaling. The convergence of these pathways further supports a multi-target, systems-level mechanism in which combined phytochemicals act synergistically to modulate metabolic homeostasis. Together, these findings support the concept that adiposity reduction and linear growth can be dissociated in pediatric obesity.

Nevertheless, these mechanistic findings should be interpreted cautiously. Network pharmacology and docking analyses are predictive tools and do not confirm direct biological activity or clinical efficacy. Experimental validation using molecular assays, endocrine markers, and relevant in vitro and in vivo models will be required to determine whether these predicted pathways mediate the observed changes in body composition and growth-related outcomes. Moreover, given that the formulation contains *Ephedra sinica*, future prospective studies should include systematic monitoring of cardiovascular and neuropsychiatric safety parameters, including blood pressure, heart rate, sleep disturbance, irritability, and appetite changes, as well as laboratory assessments of hepatic and renal function.

No adverse events were recorded in the medical records during the treatment period. Treatment adherence was monitored through regular outpatient follow-up and assessment of remaining decoction packets. However, the real-world clinical study design and lack of standardized laboratory monitoring limit definitive safety conclusions. Future prospective studies should include standardized safety monitoring, including laboratory assessments, adverse event grading, and longer follow-up, particularly because the intervention was administered to a pediatric population.

Several limitations should be acknowledged. First, this was a real-world observational study without a control group, precluding causal attribution of the observed changes to Soagambi-tang. All participants also received lifestyle guidance, including recommendations to reduce sugar intake, which may have contributed to the observed changes, particularly in body fat percentage. Because dietary intake and physical activity were not quantitatively monitored, the effects associated with Soagambi-tang could not be clearly distinguished from those of lifestyle modification. Biochemical parameters, including fasting glucose, insulin, and lipid profiles, were not routinely assessed; therefore, insulin resistance and other metabolic abnormalities could not be evaluated. Second, pubertal status was not systematically assessed, despite its substantial influence on linear growth, body composition, and endocrine function, and thus represents an important potential confounder. Third, the sample size was small (n = 25), and multiple exploratory subgroup analyses were conducted without adjustment for multiple comparisons, increasing the possibility of chance findings and limiting the robustness and generalizability of the results. Fourth, although no adverse events were documented in the available medical records, this should not be interpreted as evidence of safety. Standardized cardiovascular and laboratory monitoring was limited, which is particularly relevant because Soagambi-tang contains *Ephedra sinica*.

The daily crude-herb dose of *Ephedra sinica* was 4 g/day, which was within the previously recommended range of 2.6–6 g/day for pediatric patients aged over 2 years, adjusted according to body weight. However, adherence to established dosage guidelines alone does not definitively prove pharmacological safety. Therefore, this study cannot formally establish the complete safety profile of the intervention, though our findings reflect favorable real-world clinical tolerability. Finally, the in silico network pharmacology and molecular docking analyses provide hypothesis-generating rather than causal mechanistic evidence and require experimental validation.

Accordingly, the observed reductions in adiposity and maintenance of linear growth should be interpreted as preliminary associations rather than definitive treatment effects. Future studies should validate these findings in adequately powered prospective controlled trials with appropriate comparator groups and predefined statistical analysis plans, including adjustment for multiple testing where applicable. Systematic assessment of pubertal stage, endocrine parameters including the growth hormone–IGF-1 axis, dietary intake, sugar consumption, and physical activity would help address major sources of confounding. Prospective studies should also include systematic adverse-event surveillance and standardized cardiovascular and laboratory monitoring. In addition, transcriptomic, proteomic, and metabolomic approaches, together with experimental validation in relevant in vitro and in vivo models, are needed to clarify the biological mechanisms underlying the observed clinical associations.

## 4. Materials and Methods

### 4.1. Real-World Clinical Data Analysis

#### 4.1.1. Study Design

This real-world clinical study included children with overweight or obesity treated at a tertiary referral center in South Korea. A multi-component phytochemical formulation (Soagambi-tang), commonly used for weight management, was administered. The daily dose of Soagambi-tang consisted of *Cinnamomum cassia* bark, *Zingiber officinale* rhizome, *Ziziphus jujuba* fruit, *Glycyrrhiza uralensis* root and rhizome, *Paeonia lactiflora* root, *Ephedra sinica* Stapf herbaceous stem, *Gypsum Fibrosum*, and *Prunus armeniaca* seed (Table 2). Soagambi-tang was prepared as a water decoction at the herbal dispensary of Kyung Hee University Korean Medicine Hospital. The total amount of crude herbal materials used for one daily dose was 76 g. The herbs were decocted and concentrated to a final volume of 200 mL, which was administered orally in two divided doses of 100 mL each per day, and underwent a 6-month post-treatment follow-up evaluation. The study was conducted in accordance with the Declaration of Helsinki and was reviewed and approved by the Institutional Review Board (KOMCIRB 2026-03-015).

#### 4.1.2. Study Population

Children aged 7–16 years who first presented between May and August 2025 with insufficient height growth as the chief complaint were screened for eligibility. Participants were included if they were classified as overweight or obese according to age- and sex-specific BMI criteria, had no identifiable organic cause of overweight or obesity, received Soagambi-tang as the primary intervention, and had available pre- and post-treatment measurements of height and body weight-related parameters. Children were excluded if they had an underlying organic disease that could contribute to overweight or obesity or if their medical records were incomplete, including absence of follow-up data. In addition to Soagambi-tang treatment, all participants received lifestyle guidance, particularly focused on reducing the intake of sweets and sugar-containing foods or beverages. Safety was carefully monitored throughout the treatment period through regular outpatient visits and additional communication with parents by telephone when needed, allowing for timely assessment of any adverse events or treatment-related discomfort.

#### 4.1.3. Data Collection and Analysis

Sociodemographic and clinical data were extracted from medical records, including age, sex, height, weight, body mass index (BMI), family history, and relevant medical history (e.g., prior obesity treatment and comorbidities). To reflect real-world clinical practice, treatment-related information, including regimen and duration, was also collected. Obesity-related outcomes included changes in body weight, height, BMI, and body composition parameters such as body fat percentage measured by bioelectrical impedance analysis. BMI, height and weight z-scores and percentile were accessed in 29 April 2026 from the Baylor College of Medicine, Children’s Nutrition Research Center, Body Composition Laboratory Web Site: http://www.bcm.edu/bodycomplab/BMIapp/BMI-calculator-kids.html (accessed on 29 April 2026). Height velocity was calculated based on the 2017 Korean National Growth Charts for children and adolescents published by the Korea Centers for Disease Control and Prevention. Adverse events were evaluated through a structured audit of longitudinal outpatient medical records. At follow-up visits, patients and/or their caregivers were asked about newly developed or worsening symptoms during Soagambi-tang administration, including palpitations and other potential treatment-related symptoms, and clinically relevant findings were documented. We extracted the occurrence and type of adverse events and whether they resulted in dose modification or treatment discontinuation. Data are presented as mean ± standard deviation. Pre- and post-treatment differences were analyzed using paired *t*-tests or Wilcoxon signed-rank tests, as appropriate. Statistical significance was defined as *p* < 0.05.

#### 4.1.4. Statistical Analysis

Statistical analyses were performed to evaluate changes in anthropometric and body composition outcomes before and after treatment. Continuous variables are presented as mean ± standard deviation, and categorical variables are summarized as frequencies and percentages. Pre- and post-treatment changes in height, weight-related parameters, BMI z-score, BMI percentile, height z-score, height percentile, body fat percentage, fat mass, and lean body mass were assessed using paired *t*-tests. When the distribution of paired differences was considered non-normal or when subgroup sample sizes were small, the Wilcoxon signed-rank test was additionally applied as a non-parametric sensitivity analysis. Effect sizes for paired changes were calculated using Cohen’s d. BMI percentile categories were classified according to age- and sex-specific criteria, and changes in weight-status category, including transitions from obesity to overweight or from overweight to normal weight, were summarized as proportions. Annual height growth velocity was calculated from the observed height gain and follow-up duration, and values were compared descriptively with age- and sex-based expected growth ranges. Pearson correlation analysis was used to examine associations between changes in BMI z-score, height z-score, height, and lean body mass; Spearman correlation analysis was additionally performed to confirm rank-based associations. All statistical tests were two-sided, and *p*-values < 0.05 were considered statistically significant. Because of the exploratory nature of this study, no formal adjustment for multiple comparisons was performed; therefore, *p*-values should be interpreted descriptively.

### 4.2. Computational Analysis

#### 4.2.1. Network Pharmacology Analysis

To investigate potential mechanisms of Soagambi-tang, molecular docking and network pharmacology analyses were conducted using active compounds derived from the principal herbal components of the decoction. Candidate compounds were screened based on pharmacokinetic properties, including absorption, distribution, metabolism, and excretion (ADME). Data on Caco-2 permeability, oral bioavailability (OB), and drug-likeness (DL) were obtained from publicly available databases, including TCMSP (https://www.tcmsp-e.com/ accessed on 30 April 2026), SwissADME, and PubChem (https://pubchem.ncbi.nlm.nih.gov/ accessed on 30 April 2026). Compounds with favorable pharmacokinetic profiles (OB ≥ 30%, DL ≥ 0.18, and Caco-2 permeability ≥ −0.4) were selected for further analysis. Putative targets were predicted using SwissTargetPrediction (version 2019; http://swisstargetprediction.ch/ accessed on 30 April 2026), and pediatric obesity-related genes were retrieved from the GeneCards database (http://genecards.org/ accessed on 30 April 2026). Overlapping targets were identified using the Venny tool (https://bioinfogp.cnb.csic.es/tools/venny/ accessed on 30 April 2026) and subjected to functional enrichment analysis using DAVID (version 6.8; https://davidbioinformatics.nih.gov/ accessed on 30 April 2026) for Gene Ontology (GO) and KEGG pathway annotation. Protein–protein interaction (PPI) networks were constructed using the STRING database (https://string-db.org accessed on 30 April 2026). For disease-related targets retrieved from GeneCards, duplicate genes were removed, and targets were prioritized according to relevance scores when available. Functional enrichment results were interpreted using nominal *p*-values and/or adjusted q-values provided by the enrichment platform. Because the network pharmacology analysis was exploratory and hypothesis-generating, overlapping targets and enriched pathways were used only to generate mechanistic hypotheses rather than to establish causal molecular mechanisms.

#### 4.2.2. Molecular Docking Analysis

Candidate docking targets were prioritized according to their involvement in pediatric obesity and precocious puberty-related pathways and their centrality within the PPI network. Prior to docking, protein structures were processed using BIOVIA Discovery Studio Visualizer software (version 24.1.0.23298). Water molecules and non-essential ligands were removed, irrelevant domains were deleted when required, and polar hydrogen atoms were added. Crystal structures of representative target proteins were retrieved from the RCSB Protein Data Bank. Molecular docking simulations were performed using PyRx (version 2.5.8) with the AutoDock (version 1.2.7) Vina engine. Binding conformations and interaction sites were visualized using PyMOL software (version 2.5.8). Binding affinities with docking scores below −6 kcal/mol were considered indicative of relatively favorable predictive binding interactions.

## 5. Conclusions

In conclusion, Soagambi-tang treatment was associated with reductions in adiposity, increases in lean mass, and maintenance of linear growth in children with overweight or obesity in this real-world observational study. Network pharmacology and molecular docking analyses provided exploratory mechanistic hypotheses suggesting potential involvement of pathways related to lipid metabolism, energy homeostasis, and endocrine regulation; however, these computational findings do not establish causal mechanisms underlying the observed clinical changes. The findings should also be interpreted with caution because of the absence of a control group, the small sample size, and the potential influence of concurrent lifestyle guidance, including recommendations to reduce sugar intake, which may have contributed to changes in body composition. In addition, pubertal status was not systematically assessed, despite its substantial influence on growth velocity and body composition, and multiple exploratory subgroup analyses were performed without adjustment for multiple comparisons. Therefore, prospective controlled studies with larger sample sizes, standardized assessment of pubertal and endocrine status, careful control of lifestyle-related confounders, and predefined statistical analyses with appropriate adjustment for multiple testing are warranted to confirm these findings.

## Figures and Tables

**Table 1:** Baseline Characteristics.

Variables	Mean ± SD	n
Age (years)	10.52 ± 2.18	25
Follow up (months)	5.19 ± 1.42	25
Height (cm)	145.64 ± 13.22	25
Weight (kg)	49.00 ± 14.07	25
BMI (kg/m^2^)	23.11 ± 3.42	25
Body fat (%)	30.24 ± 4.52	23
BMI z-score	1.52 ± 0.44	25
BMI percentile	91.71 ± 7.74	25
Height z-score	0.57 ± 0.99	25
Height percentile	64.53 ± 25.48	25
Fat mass (kg)	14.93 ± 5.81	25
Lean body mass (kg)	36.12 ± 12.08	25

**Table 2:** Components of Soagambi-tang.

Component	Medicinal Part	Representative Chemical Constituent(s)
*Cinnamomum cassia*	Bark	Cinnamaldehyde
*Zingiber officinale*	Rhizome	6-Gingerol
*Ziziphus jujuba*	Fruit	Major flavonoid/triterpenoid constituents
*Glycyrrhiza uralensis*	Root and rhizome	Liquiritigenin, glycyrrhizin
*Paeonia lactiflora*	Root	Paeoniflorin
*Ephedra sinica* Stapf	Herbaceous stem	Ephedrine, pseudoephedrine
Gypsum Fibrosum	Mineral	Calcium sulfate dihydrate
*Prunus armeniaca*	Seed	Amygdalin

## Data Availability

The original contributions presented in this study are included in the article. Further inquiries can be directed to the corresponding author.

## Abbreviations

The following abbreviations are used in this manuscript:

ACACA	Acetyl-CoA Carboxylase Alpha
ADME	Absorption, Distribution, Metabolism, and Excretion
AGE-RAGE	Advanced Glycation End-products – Receptor for AGE
AKT	Protein Kinase B
AMPK	AMP-activated Protein Kinase
BMI	Body Mass Index
DL	Drug-likeness
ELOVL6	Elongation of Very Long Chain Fatty Acids Protein 6
FASN	Fatty Acid Synthase
FOXO	Forkhead Box O
GO	Gene Ontology
IGF-1	Insulin-like Growth Factor 1
KEGG	Kyoto Encyclopedia of Genes and Genomes
NAMPT	Nicotinamide Phosphoribosyltransferase
NAD^+^	Nicotinamide Adenine Dinucleotide
OB	Oral Bioavailability
PI3K	Phosphoinositide 3-Kinase
PNPLA3	Patatin-like Phospholipase Domain-containing Protein 3
PPI	Protein–Protein Interaction
PyRx	Python Prescription (Virtual Screening Tool)
SCD	Stearoyl-CoA Desaturase
SIRT1	Sirtuin 1
SIRT2	Sirtuin 2
